# Talking about intimate partner violence in multi-cultural antenatal care: a qualitative study of pregnant women’s advice for better communication in South-East Norway

**DOI:** 10.1186/s12884-017-1308-6

**Published:** 2017-04-19

**Authors:** Lisa Maria Garnweidner-Holme, Mirjam Lukasse, Miriam Solheim, Lena Henriksen

**Affiliations:** 1 Faculty of Health Sciences, Department of Nursing and Health Promotion, Oslo and Akershus University College of Applied Sciences, St. Olavs Plass, P.O. Box 4, 0310 Oslo, Norway; 2 Division of General Gynaecology and Obstetrics, Oslo University Hospital, Nydalen, P.O. Box 4950, Oslo, 0424 Norway

**Keywords:** Intimate partner violence, Communication, Culture-sensitivity, Antenatal care

## Abstract

**Background:**

Intimate partner violence (IPV) against women constitutes a major public health problem. Antenatal care is considered a window of opportunity to disclose and to communicate about IPV. However, little is known about how women from different ethnic backgrounds wish to communicate about their experiences with IPV during pregnancy in antenatal care. The aim of the present study was to explore how women from different ethnic backgrounds experienced IPV and what their recommendations were about how midwives should communicate about IPV in antenatal care.

**Methods:**

Qualitative individual interviews with eight women who had experienced IPV during pregnancy were conducted and analysed using thematic analysis. The participants were purposively recruited from three crisis shelters in South-East Norway.

**Results:**

The participants either had immigrant backgrounds (*n* = 5) or were ethnic Norwegians (*n* = 3). All participants received antenatal care by a midwife. Although none of the participants were asked about IPV during antenatal care, they wished to talk about their experiences. Most participants felt that it would be important for the midwife to make them aware that they were victims of violence. Participants offered different suggestions on how and when midwives should talk about IPV. Facilitators to talk about IPV with the midwife were a good relationship with and the trustworthiness of the midwife, information about possible negative health outcomes for the newborn owing to IPV and knowing that the midwife could help them. The main barriers to talk about IPV with the midwife were that the participants were accompanied by their husbands during antenatal care, fear that the Child Welfare Service would take away their children after disclosure and cultural acceptance of violence. Participants with immigrant backgrounds also experienced difficulties in talking about IPV owing to their limited language skills. They thought that professionally trained interpreters with experience of IPV could overcome this barrier.

**Conclusion:**

Even though none of the participants were asked about IPV in antenatal care, they offered different suggestions on how and when midwives should talk about IPV. Participants irrespective of their ethnical backgrounds perceived antenatal care as a key area to facilitate disclosure of IPV. Midwives’ communication and strategic skills to address IPV are crucial for help-seeking women. Training midwives’ skills in culture-sensitive communication might help to overcome cultural barriers to talk about violence.

**Electronic supplementary material:**

The online version of this article (doi:10.1186/s12884-017-1308-6) contains supplementary material, which is available to authorized users.

## Background

Intimate partner violence (IPV) against women constitutes a major public health problem [1, 2]. According to the definition of the World Health Organization (WHO), IPV may include physical aggression, sexual coercion, physiological abuse and/or controlling behaviours by current or former partners [2]. Estimates of the global prevalence of IPV vary, partly because of differences in the definition, context, material and methods used when examining violence [3–5]. In a recent meta-analysis of IPV during pregnancy from 92 studies from 23 countries, the average reported prevalence of emotional abuse, physical abuse, and sexual abuse was 28.4, 13.8, and 8.0%, respectively [5]. In Norway, the prevalence of IPV during pregnancy was reported to be 1–5% [3, 6–9]. These numbers are comparable with those of a new longitudinal cohort study from Sweden, in which 2.5% of 1573 women reported violence during pregnancy [10].

It is recognised that violence has an adverse impact on women’s physical, reproductive and mental health, including pregnancy complications [2]. Violence in pregnancy is associated with reproductive coercion, unintended/rapid repeat pregnancy, poor maternal weight gain, hyper-emesis, antenatal hospitalizations, miscarriage, vaginal bleeding, mode of delivery, preterm birth and maternal mental health problems [11]. IPV can have a significant impact on women’s parenting abilities, which, in turn, can compromise their children’s development [12]. Known risk factors for violence against women are being young, single or drug and alcohol consumers and having low economic status and a former history of abuse [13, 14]. Although immigrant women are a heterogeneous group, factors related to their migration context, including economic insecurity, language barriers, family separation, social isolation and discrimination, may make them more vulnerable to IPV [15].

Antenatal care is considered a window of opportunity to communicate about IPV, as the repeated visits during pregnancy allow for the development of trust and confidence between the pregnant woman and the healthcare provider [16]. In Norway, almost every pregnant woman attends antenatal care, a free and well-integrated part of the public health system [17]. Antenatal care is shared between general physicians (GP) and midwives in primary health care. A woman can choose whether she will visit the GP or the midwife or both alternately. The overall goal of antenatal care is to ensure the well-being of the mother and fetus and to discover complications. Following recommendations from the WHO and the National Institute for Health and Care Excellence (NICE) guidelines [18, 19], in 2014 new guidelines were introduced by the Norwegian health authorities to encourage health professionals to routinely ask women about their experiences of IPV. Thus, midwives can play a major role in the identification of IPV and subsequent care of women experiencing IPV. However, previous studies have suggested that health professionals find it challenging to communicate about sensitive issues like violence, especially when faced with a multicultural patient population [20–22]. Even though it is acknowledged that health communication should be culturally sensitive [23–25], studies indicate that health professionals may lack the communication skills [20].

Previous researchers have investigated how pregnant women experience communication about IPV in antenatal care [26–28]. Edin et al. identified that failed interactions with midwives, lack of policies and pregnant women’s strategies to keep up a front to hide the violence from others were barriers to communicating about violence. Even though studies including pregnant women of different backgrounds are sparse, there is some evidence that immigrant pregnant women may have other needs regarding communication about IPV with their midwife than women of the majority ethnic group [29]. While midwives in Norway are strongly encouraged to routinely ask women about their experience of IPV, there have been no studies to explore women’s opinions of this practice. Therefore, the aim of our study was to investigate pregnant women’s experiences with and recommendations to communicate about IPV in Norwegian antenatal care. Women with different ethnical backgrounds were included to have a study sample that may represent the variety of women who visit antenatal care at Norwegian Mother and Child Health Centres (MCHCs).

## Methods

### Recruitment

Women who had experienced violence during pregnancy were purposively recruited from three crisis shelters in South-East Norway. Employees at these crisis shelters recruited women with children who previously or currently lived at the shelter. Participants received verbal and written information about the study. They were told about the purpose of the study and that they would not be asked questions about their personal violence experience. Recruitment was carried out until a richness of individual cases was reached [30].

### Data collection

Individual interviews with eight pregnant women who had experienced different types of IPV during pregnancy were conducted. An explorative qualitative approach with individual semi-structured interviews were chosen to gain a deeper insight into personal experiences with a sensitive topic [31]. The interviews took place in a private room at the crisis shelters between July and September 2016. The interviews followed a semi-structured interview guide (Additional file 1). The main themes in the interview guide were (1) background information about the participants’ antenatal care; (2) experiences of communication about IPV in antenatal care; (3) participants’ advice for how to communicate about IPV in antenatal care; (4) communication material; and (5) motivation to disclose violence. The authors LH and MS performed the interviews together, and each interview lasted approximately 45–60 min. One interview was conducted with the help of a certified and experienced interpreter, recommended by the crisis shelter where the woman was interviewed. Interviews were audio-taped and transcribed verbatim by MS. Two other authors, LH and LGH, compared the audio tapes randomly with the transcripts to ensure the accuracy of the transcription process. The interviews followed the Helsinki Protocol and WHO’s guidelines for researching violence against women [32, 33]. To fulfil the ethical principles of these guidelines, the interviews were conducted at crisis shelters to grant participants safety, and participants were informed that they did not have to answer questions that they were uncomfortable with. In addition, staff at the centre were aware of the content and purpose of the interviews and were available for counselling afterwards.

### Data analysis

The analysis of the interviews was guided by thematic analysis, according to Braun and Clarke [30]. The analysis was approached inductively and included (1) familiarising with the data by repeated reading of each informant’s transcripts; (2) generating initial codes (words or short phrases in the transcripts) which were relevant to understand the meanings individuals attach to their experiences; (3) organizing codes into sub-themes; (4) arranging sub-themes into overarching themes; (5) defining and naming the themes. The authors MS and LGH conducted the analysis. The potential themes were discussed with the other authors to improve the credibility of the findings.

## Results

### Characteristics of study participants

To capture a variety of experiences, informants with varying places of birth and years of residence in Norway were recruited (Table 1). All participants had given birth to at least one child in Norway. All participants had received antenatal care through a midwife. The time since the participants received antenatal care was 1–16 years.

**Table 1:** Informant characteristics (*n* = 8)

Participant	Country of birth	Years of residence in Norway	Parity and age of the youngest child (years)
1	Iraq	11	2 (4)
2	Turkey	12	2 (10)
3	Norway	–	3 (10)
4	Norway	–	1 (16)
5	Pakistan	?^a^	3 (?^a^)
6	Poland	10	3 (2)
7	Norway	–	1 (3)
8	Spain	3	3 (1)

^a^The participant did not want to provide this information

The analysis resulted in four themes representing participants’ experiences with and recommendations for communicating about IPV in antenatal care: *Experiences with communication about IPV* describes how participants experienced not being asked about violence during antenatal care, even though they considered antenatal care as a good arena to talk about it. *Advice on how midwives should talk about IPV* illuminates how participants recommend midwives to talk about IPV. *Facilitators to talk about IPV* elucidates several aspects that women considered important to feel comfortable to disclose violence in antenatal care. In contrast, *barriers to talk about IPV* summarizes women’s perceptions about what would make conversations about IPV difficult.

### Women’s experiences with communication about IPV during antenatal care

Four sub-themes emerged in this theme: (1) the midwife did not ask about violence; (2) antenatal care was a good arena; (3) lack of facilitators to talk about IPV and (4) midwives were perceived as powerless. None of the participants were asked about IPV during antenatal care, including two participants who received antenatal care after the implementation of the new 2014 guidelines that instructed midwives to routinely ask all pregnant women about IPV. Seven out of eight women considered antenatal care by a midwife as a good arena to disclosure their experiences with violence, as expressed by a participant who attended antenatal care at an MCHC during all of her three pregnancies:

*“…Violence, that was never a topic. It wasn’t even mentioned with a single word. I think that was what I’ve missed, because you are caught and can’t escape.”* (participant 3)

However, participants perceived that was absolutely nothing that facilitated communication about IPV during antenatal care. They hoped that the midwife would observe the signs that their partner was violent, especially when the partner joined the consultation. They expected the midwife to be someone they could rely on and who could make them feel safe and provide guidance.

A participant who received antenatal care by a midwife two times and who also worked as an interpreter in antenatal care felt that some midwives started to ask about violence after the implementation of the new guidelines. However, she felt that midwives were powerless when women disclosed violence:

*“They appear to be very powerless when they meet a woman who has experienced violence during pregnancy.”* (participant 6)

### Women’s advice on how to communicate about violence in antenatal care

We identified the following sub-themes in this theme: (1) starting the conversation about IPV; (2) importance of explaining what violence is; (3) communicating about IPV toward the end of pregnancy; (4) organising antenatal care and (5) providing helpful materials to talk about IPV. Participants provided individually varying advice on how the midwife could start a conversation about women’s experiences of violence. For instance, one women from Poland who received antenatal care 2 years previously in Norway stated that it would be important for the midwife to explain why she asks about violence:

“…*If she would ask, she should explain to me why she asks. Because, is she going to do something about it, or is she just curious? (…) I think that it is wrong that they say that they have to ask because it says so in the guidelines. (…) For me, that sounds like she just wants to get over with it.*” (participant 6)

Some women, independent of their ethnic background, recommended that the midwife should ask the women directly about whether they have experienced violence:

“…*For me, it’s best directly, actually. Because if someone starts to ask around it, I’ll answer, but then I think, “what is she really getting at with all this*?” (participant 6)

Others advised that midwives should start communicating about violence more openly, such as by asking about women’s understanding of violence:

“…*Yes, she should start for example with the question, like, what is violence for you? I think we women, we don’t know the difference between… hmm, what we have to tolerate or not. I mean that we, by the time we recognize the violence, the trouble has already been lasting for too long*.” (participant 8)

This opinion was shared by another woman, who recommended that midwives should explain the different forms of violence to make women aware that they were victims. A Turkish woman who had experienced violence in both of her pregnancies said the following:

“…*Maybe women need more information about what violence is? I didn’t know that physically and psychologically violence are two different things. (…) I thought that violence means to beat, really beat. Not only once or twice*.” (participant 2)

Participants suggested that midwives should talk about violence in the middle or toward the end of pregnancy, either because there was too much information about other health-related issues in the beginning of the pregnancy or because they felt that it was important to first build trust in the midwife:

“…*I think in the middle of pregnancy. When you are used to being pregnant. I think it could be too scary in the beginning.”* (participant 4)

Independently, participants stated that it would be important for the midwife to establish strategies for how to communicate with women who disclose experiences of violence.

Women recommended that the guidelines for antenatal care should include one private session in which a woman can meet the midwife alone to have the opportunity to talk about violence. Some participants expected the midwife to teach them methods to increase their own safety in this session. One woman who recently escaped from her violent husband and currently lived at the crisis shelter described her own safety behaviours and thought that it would be very important for other women to receive similar information:

“…*I’ve always had a packed bag in my car, for both myself and my children. I’ve always had our passports with me, hidden in the bag. And I’ve written a personal diary… so that you afterwards remember when and what happened*.” (participant 8)

Others stated that their husbands would not allow them to visit antenatal care if they were to find out that the midwives were asking them about violence. In particular, participants living in rural areas would prefer to get information about violence in group sessions at the MCHC to ensure anonymity. For instance, one ethnic Norwegian participant residing in a rural area thought that it would be difficult to talk to the midwife about her experiences because everybody knows everybody in her hometown.

Participants had several suggestions for helpful information material about IPV. For example, women asked for materials that explain step-by-step how they could escape from their violent partner:

“*…But I think, if we should have a map, like, (…) The escape route. (…) For me it’s still not really clear how the escape route is? But there is an escape route and we should know it and we should make it very clear. Like a poster or whatever. Like, your escape route is that: You can contact this, and you have this here and then the next step and the next step and you are out.”* (participant 8)

Others recommended a movie that could be shown at the MCHC. Several women wished for written information material, like a contact card for the shelter, in the waiting room at the MCHC. Even though several participants said that they could never bring brochures or other written material about IPV home, they mentioned that it would help to read the information at the MCHC to get help without disclosing IPV. Accessing information about IPV from the Internet was not an option for most of the participants, because their partners checked their Internet search histories.

### Facilitators to talk about IPV in antenatal care

Participants were asked about what would motivate and enable them to talk about violence with the midwife. The most strongly emerging sub-themes were a good relationship with and trust in the midwife. In particular, participants with immigrant backgrounds experienced loneliness and outlined the importance of the midwife in their life. For example, a woman originating from Turkey and who had lived in Norway for more than 12 years said the following:

“…*When you live with a person and you are not allowed to have other persons in your live… You have nobody. For some, the midwife is the only person they can meet, and that’s why she gets very important.”* (participant 2)

Further sub-themes within this theme were as follows: (1) the midwives’ knowledge about violence; (2) information about health consequences; (3) the consequences of disclosing violence and (4) the use of a professional interpreter. Several participants were afraid that their midwife did not have enough knowledge about violence. Many participants thought that information about the consequences of IPV on their unborn child and their own health would have motivated them to seek help from the midwife. One woman who had experienced violence by her husband during three pregnancies said the following:

“…*If I would have known what could happen to the baby when the mother is stressed and impatient, and that it’s not only things you eat that influences the health of your child… I think I would have talked about it earlier.”* (participant 6)

Participants said that it would be easier to talk about violence if they would be sure that their disclosure would result in a helpful response. An ethnic Norwegian woman explained:

“…*You don’t dare to talk about it if you are not sure whether or not it would lead to real consequences.”* (participant 4)

In the same context, participants said that it would be very important for the midwife to inform them about their legal rights related to violence. One participant who migrated to Norway from Pakistan said that she was not aware that violence is forbidden in Norway:

“*I was not aware that violence was forbidden in Norway, that there should not be violence, and that it would be against the law.*” (participant 5)

Some participants with immigrant backgrounds experienced language difficulties during their consultations and thought that this made it difficult to talk about violence. They also experienced challenges in communicating with their midwife via an interpreter, and they thought that the use of professional interpreters that have experience with violence would facilitate the conversation with the midwife.

### Barriers to talk about IPV in antenatal care

The following four sub-themes represented participants perceived barriers to talk about IPV in antenatal care: (1) being accompanied by the husband; (2) fear of the Child Welfare Service; (3) talking about violence not being accepted in their culture and (4) fear that nobody would believe them. All participants were accompanied by their husbands for most antenatal care consultations, making it impossible for them to talk about violence with the midwife:

*“There has never been an opportunity to talk about violence, since my husband always has accompanied the consultations.”* (participant 5)

Some were also afraid to talk alone with the midwife about violence, because the midwife might mention it accidentally the next time the husband attended the consultation again. Others stated that their husband would not allow them to visit the antenatal care on their own if they knew that the midwife would ask about violence at this visit, as expressed by the following participant:

*“Actually, I think that my husband was so afraid that somebody would get to know what was going on that he would refuse me to visit the midwife on my own.”* (participant 4)

The participants stated that they would not disclose violence to their midwife since they were afraid that the Child Welfare Service would take their children from them. In this context, one participant from Spain mentioned that she was very surprised that there was so little focus on violence in Norway, and she felt that women’s rights were very different from those in her country of birth. For instance, she was surprised that she had to move in to the shelter whereas her husband could stay at home.

Some participants with immigrant backgrounds felt that it could be difficult to talk about violence with their midwife, because one did not talk about violence in their culture. For example, one Iraqi woman who had lived in Norway for 11 years expressed:

“…*Our culture is a little bit strict about it… to tell that the husband is mean against his wife.*” (participant 1)

One women was also afraid that the midwife might not believe in her. This problem was especially related to psychological violence:

“…*With physical violence, you can take a picture and you have proof, but, with psychological violence?”* (participant 3)

The same participant was also unsure about whether or not the midwife could help them.

## Discussion

This study showed that women wished to talk about their experiences of IPV in antenatal care. Most participants noted that it was important for the midwife to make them aware that they were victims of violence. Participants offered different suggestions on how and when midwives should talk about IPV. Facilitators to talk about IPV with the midwife were a good relationship with and the trustworthiness of the midwife, provision of information about possible negative health outcomes to the newborn owing to IPV and knowing that the midwife could help them. The main barriers to talk about IPV with the midwife were that the participants were accompanied by their husbands during antenatal care, fear that the Child Welfare Service would take away their children after disclosure and cultural acceptance of violence. Participants with immigrant backgrounds experienced difficulties in talking about IPV owing to limited language skills and the thought that professionally trained interpreters with experience with IPV could overcome this barrier.

This is the first study to investigate women’s experiences with communication about IPV in antenatal care in Norway. The women in our study were in favour of enquiring about IPV in antenatal care. This is in line with results from qualitative and quantitative studies in several other countries [28, 34–36]. Similar to the results in these studies, our participants thought that it would be important for professionals to raise their awareness of the fact that they were victims of violence. For instance, Chang et al.’s qualitative study of Australian women described that screening by a sensitive provider made them aware that they were victims and motivated them to reach out for help [28]. However, there are few comparable studies among immigrant women [29]. A qualitative study of Somali-born refugees in Sweden revealed that midwives’ questions about violence were met with hesitance [29]. In contrast, women from immigrant backgrounds in our study said that they were lonely and that the midwife was the only person they could talk to about their violent husband. Nevertheless, participants in both studies said that they could open up to the midwife if the midwife explained confidentiality and the links between violence and health.

Another important motivation to talk about violence in antenatal care was that the majority of our participants believed that the midwife could help support them in their situation or help them come out of it. Edin et al. (2010) conducted a comparable qualitative study among women who had been subjected to severe IPV during pregnancy in Sweden. In contrast to our participants, the women in their study had actually been asked about IPV during antenatal care. The authors found that three out of nine women chose to disclose violence to the midwife, but only one said that doing so was helpful. Women perceived midwives as being mainly responsible for the somatic side of pregnancy rather than as a potential resource to help them with difficulties [26]. Few evidence-based interventions have investigated whether or not women’s disclosure of violence helps them to reduce or escape from their experiences of violence. Miller et al. designed a treatment model that combines mental health and advocacy services [37]. This model was effective in reducing violence and the re-victimization risk for women exposed to IPV. However, it has not yet been tested among pregnant women, and more knowledge is necessary for devising strategies to prevent IPV during pregnancy.

Even though our participants wished to talk about violence with their midwives, they mentioned several barriers that could prevent them from disclosing IPV. A focus group study among mainly African-American women in the United States found that women often chose not to disclose violence [38]. As in our study, fear of the partner was one of the main barriers to disclosure. The Norwegian guidelines for antenatal care suggest that antenatal care should include one private session with the midwife to talk about violence. However, there is a dilemma, because some of our participants said that they would not be allowed to come to such a consultation if the husband knew or suspected that women would be asked about violence when on their own.

Our study participants believed that freely available information about IPV at the MCHCs could help them even though their husbands accompanied them during antenatal care. Some of our participants asked about information on violence on the Internet; however, others could not use the Internet because their partners checked their search histories. A study of pregnant patients in obstetric clinics in Australia compared women’s preferences of in-person versus computer screening for IPV [39]. Participants were more likely to disclose IPV via a computer mainly owing to anonymity and easier questions. Bacchus et al. conducted a technology-based IPV intervention in perinatal home visitation among women experiencing IPV in the United States. An intervention strategy on a computer tablet was perceived as a safe and confidential tool for initiating discussions about IPV with health professionals, assisting women in enhancing their safety and exploring help-seeking options [40]. As most pregnant women in Norway attend antenatal care at MCHCs, these would serve as a good arena to test a technology-based intervention to disclose violence.

In line with other studies, our interviews indicated that midwives’ skills and strategies to communicate about violence might overcome women’s barriers to talk about their experiences with violence [35, 41, 42]. Our study participants thought that midwives could become better at communicating about IPV if they would have more knowledge about it. For instance, midwives should know about the different forms of violence, and they should provide information about the possible health consequences of IPV for the baby as well as women’s options to get help. These findings are in line with previous studies, where women were more likely to talk about IPV when the communication focused on the provision of more knowledge about violence, rather than the disclosure of violence [28, 43].

Furthermore, our study illustrated that participants preferred different communication strategies and styles about IPV. Some women suggested that midwives should directly ask about violence, whereas others preferred to first receive information about it. Previous authors have studied different communication strategies that motivate women to talk about IPV in antenatal care [26, 41]. O’Doherty studied women’s comfort in disclosing IPV in a clustered randomized trial about women’s evaluation of abuse and violence in general practice. Spending enough time with a woman, simply facilitating unhurried communication and a patient-centred care approach were identified as important factors for improved communication [41]. Previous research on multi-cultural health communication indicates that a patient-centred open communication style may be novel for immigrants from African and Asian countries who are used to another health-professional-client relationship [44–46]. Possibly owing to our small study sample, we did not observe preferences in midwives’ communication styles related to cultural backgrounds in our participants.

Our study suggested that communication about IPV should be individualized in relation to a woman’s circumstances and varying informational needs. Individually tailored communication may be defined as any combination of strategies and information intended to reach one specific person, based on characteristics that are unique to that person, related to the outcome of interest and derived from an individual assessment [47]. Our study indicated that the acknowledgement of ethnic and cultural differences could be important aspects for successful individualized communication about IPV. Participants from immigrant backgrounds said that violence was accepted in their culture. Furthermore, they thought that it was important for the midwife to make them aware of the abnormality of violence and that they were victims. Foronda stated that culture sensitivity improves health communication [38]. Culture sensitivity involves health professionals’ knowledge of cultural differences, consideration of their clients, understanding of cultural values, respect for culture and language and tailoring of communication to meet the client’s needs [48]. Midwives’ knowledge of cultural differences and consideration of women’s life situations may help overcome these barriers. Spangaro et al. studied Australian Aboriginal women’s decision to disclose IPV during pregnancy. They found that ‘cultural safety’ was central to indigenous women’s decision to disclose violence, and they identified processes for creating safety [49]. Cultural safety implied building trust in the relationship followed by providing enough time to talk about violence, which was also found in our study. Tailoring of communication to meet the client’s needs may also involve the use of professional interpreters, as suggested by our participants. However, future research should investigate the influence of an interpreter on the communication about violence in antenatal care.

### Limitations

The aim of this study was to get a better understanding of experiences with and recommendations for communication about violence in antenatal care among pregnant women from different ethnic backgrounds. We found some indications that midwives should acknowledge ethnic and cultural differences related to the women’s background; however, the study sample was too small to draw conclusions for the immigrant population at large. Future research should be conducted among specific immigrant groups. Even though the researchers aimed to solve linguistic misunderstandings during the interviews, collecting data across languages and cultures was challenging, and language difficulties might have influenced the quality and interpretation of the interviews. The use of an interpreter may imply an additional step of interpretation, and thus, it might have influenced the analysis of the interviews [50]. Interviews were transcribed immediately after the interview session, and they were proofread by both researchers conducting the interviews to ensure the accuracy of the transcription. In this study, researchers had different educational backgrounds, however, all decisions and agreements were shared and discussed until consensual validation was achieved. We faced problems in recruiting women who received antenatal care after the recent introduction of new guidelines for antenatal care at our recruitment sites. Thus, for some participants, many years had passed since they received antenatal care, and their recall of their communication about IPV during pregnancy might have been limited. Future studies should try to recruit women who have received antenatal care recently and include discussions about their experience of violence.

## Conclusions

Even though none of the participants were asked about IPV during antenatal care, this study found that pregnant women of different ethnic backgrounds want to talk about IPV with their midwives. Women recommended that midwives should explain why they ask about violence, before making them aware of the different forms of violence. Trust in the midwife was essential for women to disclose IPV. Trust takes time to build. Conversations about violence should therefor be scheduled in the middle or toward the end of pregnancy. The provision of written information about where to find help could help women who are afraid to reveal their experiences of violence. Participants’ recommendations about how to communicate about violence in antenatal care indicate that the acknowledgement of ethnic and cultural differences could be important aspects for successful individualized communication about IPV. Training midwives’ skills in culture-sensitive communication might help to overcome cultural barriers to talk about violence.

## Additional file

Additional file 1: Interview guide; Description of data: English language copy of the interview guide used to direct the semi-structured interviews in this study. (DOCX 16 kb)

## Abbreviations

GP: General Physicians
IPV: Intimate partner violence
MCHCs: Mother and Child Health Centres
NICE: National Institute for Health and Care Excellence
WHO: World Health Organization
