# Supplementary material for: Talking about intimate partner violence in multi-cultural antenatal care: a qualitative study of pregnant women’s advice for better communication in South-East Norway
Source: BMC Pregnancy Childbirth. 2017 Apr 19;17:123. doi: 10.1186/s12884-017-1308-6 (PMC5395889; doi:10.1186/s12884-017-1308-6)
Supplement: Additional file 1: — Interview guide; Description of data: English language copy of the interview guide used to direct the semi-structured interviews in this study. (DOCX 16 kb) [file 12884_2017_1308_MOESM1_ESM.docx]

# **Interview Guide**

Introduction: Thank you that you participate in this interview. We are going to start with some background information about you. Everything you tell us will be confidential and we won’t record your name.

1. **Background information**

How many children do you have?

Did you visit the midwife and /or your GP for your antenatal care?

1. **Experiences of communication about violence in antenatal care**

Did anybody talk to you about violence during your pregnancy? If yes, who did? How did you feel about that?

How was the topic approached? Did the topic come up several times?

Do you think that antenatal care is an appropriate arena to talk about violence?

1. **Participants’ advice for how to communicate about IPV in antenatal care**

How do you prefer that your midwife/GP talks about violence?

What is your advice for how violence in antenatal care should be approached?

How can the midwife/GP build trust?

1. **Communication materials about IPV**

Do you have any examples of useful information materials midwifes/GPs could provide?

What kind of offers where relevant/available for you?

Was there anything you’ve missed?

1. **Motivation to disclose violence**

What do you think motivates women to seek help?

What was your motivation to seek help?

Do you have any examples for strategies for midwives to talk about violence?

Do you have any examples of things you can do to increase your safety? Any strategies?
